# Acute Stress-Induced Changes in the Lipid Composition of Cow’s Milk in Healthy and Pathological Animals

**DOI:** 10.3390/molecules28030980

**Published:** 2023-01-18

**Authors:** Yaiza Garro-Aguilar, Roberto Fernández, Silvia Calero, Ekaterina Noskova, Marina Gulak, Miguel de la Fuente, Albert Adell, Edurne Simón, Urko Muzquiz, Diego Rodríguez-Piñón, Egoitz Astigarraga, Gabriel Barreda-Gómez

**Affiliations:** 1 Research and Development Department, Amaltea Research, 48940 Leioa, Spain; 2 Department of Pharmacy and Food Sciences, University of the Basque Country UPV/EHU, 01006 Vitoria-Gasteiz, Spain; 3 Research and Development Division, IMG Pharma Biotech, 48160 Derio, Spain; 4 Instituto de Biomedicina y Biotecnología de Cantabria IBBTEC-CSIC, 39011 Santander, Spain; 5 Cruz Roja Hospital, 48013 Bilbao, Spain; 6 Experimental Ophthalmo-Biology Group, Department of Cell Biology and Histology, University of the Basque Country UPV/EHU, 48940 Leioa, Spain; 7 Welfaretracker S.L., 31500 Tudela, Spain

**Keywords:** lipidomic, milk, biomarkers, lipid metabolites, animal welfare

## Abstract

Producers of milk and dairy products have been faced with the challenge of responding to European society’s demand for guaranteed animal welfare production. In recent years, measures have been taken to improve animal welfare conditions on farms and evaluation systems have been developed to certify them, such as the Welfare Quality^®^ protocol. Among the markers used for this purpose, acute phase proteins stand out, with haptoglobin being one of the most relevant. However, the diagnostic power of these tools is limited and more sensitive and specific technologies are required to monitor animal health status. Different factors such as diet, stress, and diseases modify the metabolism of the animals, altering the composition of the milk in terms of oligosaccharides, proteins, and lipids. Thus, in order to study oxidative-stress-associated lipids, a collection of well-characterized milk samples, both by veterinary diagnosis and by content of the acute stress biomarker haptoglobin, was analyzed by mass spectrometry and artificial intelligence. Two lipid species (sphingomyelin and phosphatidylcholine) were identified as potential biomarkers of health status in dairy cows. Both lipids allow for the discrimination of milk from sick animals and also milk from those with stress. Moreover, lipidomics revealed specific lipid profiles depending on the origin of the samples and the degree of freedom of the animals on the farm. These data provide evidence for specific lipid changes in stressed animals and open up the possibility that haptoglobin could also affect lipid metabolism in cow’s milk.

## 1. Introduction

Cattle are the main producers of milk for human consumption, as well as for the production of dairy products in the European Union (EU) and in Spain [1]. Due to the high energy supply, the contribution of essential minerals and vitamins that it provides and the high-quality protein that it contains, bovine milk is a basic, complete, and balanced food [2]. Regarding the composition of cow’s milk, it is composed mostly of water, constituting around of 87% of the total. It also contains 4–5% of lactose, around 3% of protein, 3–4% of fat, 0.8% of minerals, and 0.1% of vitamins [3,4]. Milk fat contains thousands of lipid species [5]. Precisely, milk fat is composed of three classes of associated substances: neutral lipids, which are mostly triacylglycerides (TAG) (98%); polar lipids, mainly phospholipids of a complex nature (1%); and unsaponifiable substances (<1%) [5,6,7]. TAGs are the most abundant lipid species of milk fat and are an essential energy source for humans [5]. Their structures have a great influence on the physicochemical properties of milk fat, the quality of dairy products, and milk´s absorption efficiency in humans [8,9,10,11]. They are synthetized from around 400 different fatty acids (FAs) [12]. This factor makes cow´s milk contain the most complex fat of other natural fats [4]. Due to its importance, Giannuzzi et al. observed in a recent study that FA fingerprint could be a cow´s metabolic status indicator [13].

Nevertheless, milk fat also contains minor components such as glycerophospholipids (GPL), sphingolipids (SL), and glycolipids (GL). These are the main components that constitute milk fat’s globule membrane, which is the responsible for ensuring the stability of the oil in water (o/w) emulsion of milk [14,15]. Furthermore, in a recent study, researchers found that some glycerophospholipid species present in milk may be used as potential biomarkers for cow’s health [16].

In addition to potential lipidic biomarkers, there are also protein biomarkers, such as acute phase proteins (APP), for which the blood concentration varies in response to various inflammatory processes. APPs are classified into two groups, positive and negative, depending on whether their concentration increases or decreases in animals´ blood for animals suffering from a disturbance of their immune system [17,18,19]. Haptoglobin is the APP that suffers greater change in cattle´s milk and serum, and its concentration increases between 10 to 100 times with respect to the basal level. Haptoglobin presence in blood and milk has been frequently associated with cows´ different pathologies, determining that it is a non-specific marker of disease, inflammation, or stress [18,20]. This has aroused interest in the dairy industry, since the determination of haptoglobin could become a biomarker of well-being and, therefore, of milk quality [20,21,22,23].

Milk’s composition is not always constant as it continuously undergoes changes. The causes for these changes are numerous and with a relative weight, yet to be determined. Some of the most relevant are genetics, animal´s feed, the state of the lactation cycle and accumulated lactations, and the physiological state of the animals (pathologies or animal welfare) [2,7,24]. Specific actions, such as free access to pasture, increase lipid synthesis [25]. For example, due to pasture feeding, concentrations of unsaturated fatty acids, including conjugated linoleic acid (CLA), α-linolenic (LNA, 18:3n-3), and oleic acids, could be improved in milk [26]. On the other hand, due to the genetics of the animals, a higher incidence of certain diseases can be found compared to others, with the most common diseases being mastitis, lameness, metritis, and displacement of the abomasum [27]. Between them, mastitis is the most common disease, with an incidence that reaches up to 54.5% during the lactation period and that can also occur during the dry period [28]. The mastitis process has a negative impact on different aspects as it causes a decrease in milk yield, requires the removal of animals from the productive routine, and involves treatment and prevention costs and changes in milk composition that affect its quality [29]. Animals with a high milk yield have a greater predisposition to develop mastitis and suffer greater losses in milk production than those with a lower production [30].

Evaluation systems that integrate all of the factors that influence the welfare state of animals are complex to elaborate, and in the attempt to achieve this, hundreds of evaluation systems have been developed, with different strengths and weaknesses. The most widely accepted and applied programs in the respective territories are the European Welfare Quality^®^ Assessment Protocol for dairy cattle (WQ), applicable in the European Union, the Dairy Farmers Assuring Responsible Management Program (FARM), applicable in the United States, and the Code of Welfare: Dairy Cattle (The Code), applicable in New Zealand. These three programs were created according to the most important needs and factors in each type of production [31]. The Welfare Quality^®^ project is the largest cross-sectional study in the area of well-being developed by the European Commission to date and includes research from forty European and four Latin American research centers [32]. Obtaining a good result in the audits of this protocol grants a widely recognized recognition of quality, but it is not a mandatory regulation, even if it comes from studies financed by the European Union [31,33].

Hence, the aim of this work is to demonstrate that the acute stress protein haptoglobin is related to certain lipids in cows’ milk, supporting its use as a biomarker of stress and inflammatory and pathological processes. It is estimated that it is possible to identify subclinical or asymptomatic processes in the early stages of the course of a disease through the detection of haptoglobin in milk and that these processes involve a specific and identifiable alteration in the composition of milk´s fat. Despite the importance of FA in cow´s milk composition, due to the technology we used (MALDI) to detect possible biomarkers we have focused on GPLs, because of their abundance in bovine milk and their sensibility and precision to the mentioned technology. In the case of being able to identify these molecules, it would be possible to determine new biomarkers for the early detection of pathologies in dairy cattle.

## 2. Results

### 2.1. Identification of Lipid Compounds Positively or Negatively Correlated with Milk Haptoglobin Concentration

**Figure 1:**
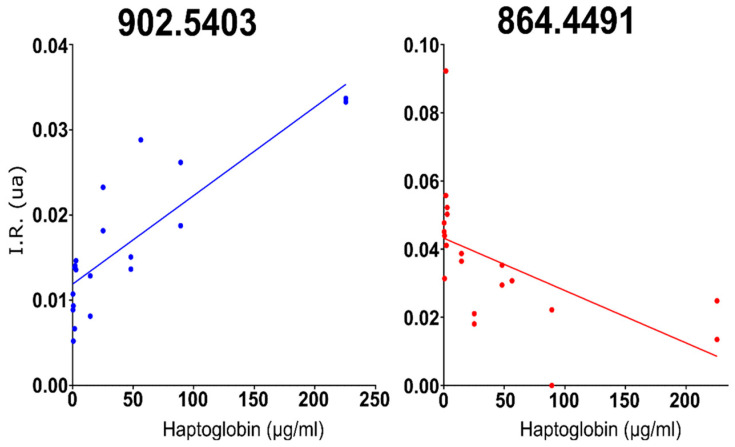
The two peaks with the best positive and negative correlations to haptoglobin concentration in milk: 902.5403 (in blue) and 864.449 (in red).

After determination of haptoglobin concentration by ELISA (Table A1), a set of milk samples was studied by mass spectrometry using an Autoflex III (Bruker, Billerica, MA, USA) supplied with MALDI source to find out whether there is a correlation between any lipid and haptoglobin concentrations. Several peaks displayed relatively high correlations with haptoglobin concentrations (Table 1). Lipid species were assigned to each *m/z* according to Liu et al.´s article “Comprehensive Characterization of Bovine Milk Lipids: Phospholipids, Sphingolipids, Glycolipids, and Ceramides” [34], as has previously been carried out in other works [35]. Figure 1 shows two notable examples of positive and negative correlations. 

### 2.2. Estimation of the Prevalence of Haptoglobin in Healthy Animals, Pathological Animals, and in the Milk Tanks of Different Farms

**Figure 2:**
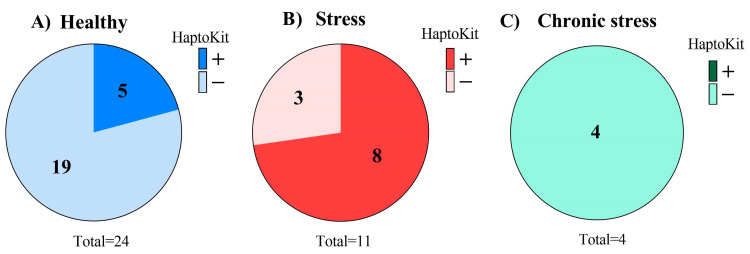
Results of the Haptokit analysis on different milk samples. (**A**–**C**) Pie charts of the Haptokit results. The 39 animals were distributed into three groups, one with healthy animals (**A**), one with acutely stressed animals (**B**), and one with animals suffering from chronic stress (**C**).

The assayed animals (*n* = 39) were distributed into three groups, one group with healthy animals (Figure 2A), another group with stressed animals (Figure 2B), and another group with animals suffering chronic stress (Figure 2C). The results of individual cows obtained in the Haptokit test for the detection of haptoglobin can be seen in Table A2. The results of each group obtained in the Haptokit test are shown in Figure 2. A higher prevalence of positive results in the Haptokit test was observed in pathological animals than in the other groups, with 50% (7/14) of the cows being positive. In the group of healthy animals, 24% (6/25) of the animals obtained a positive result in the test. Finally, all of the animals suffering chronic stress obtained a negative result in the Haptokit test, as haptoglobin is an acute phase protein.

To obtain additional information about the farms, haptoglobin detection was also carried out in the milk tanks of all of the farms (four farms in Aragón and five in Navarra) (Table 2). Two positive results were obtained in the milk tanks, which was of great interest due to the high capacity of the tanks.

In the case of the Fitero farm, they obtained a “good” rating in the last Welfare Quality audit (Table A3). On the other hand, Alfajarín farm is smaller than Fitero farm, with it containing 70 animals. This means that with a lower number of animals with haptoglobin in their milk, this manifests itself in an increase in the mixture of the entire farm. The weight of each individual’s contribution is greater in a smaller herd.

### 2.3. Lipidomic Analysis of the Milk Samples

The lipid profiles of the milk samples were obtained by mass spectrometry. Two spectra were made for each animal’s milk sample and also for the samples that were extracted from the milk storage tank.

**Figure 3:**
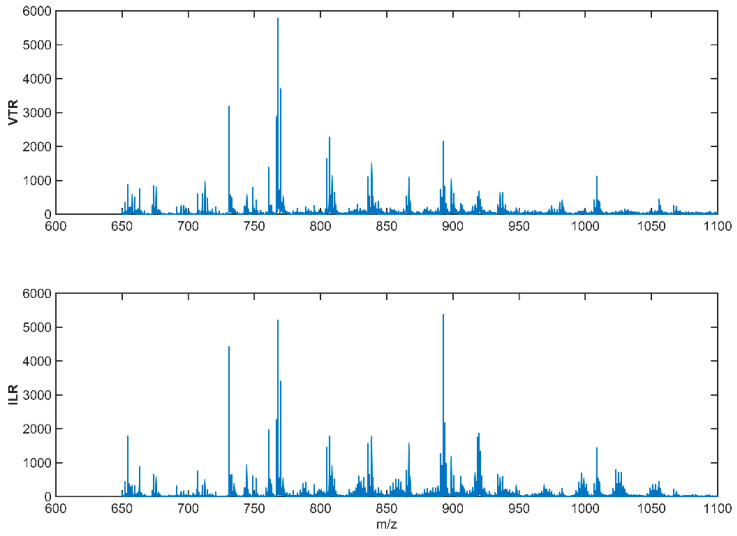
Normalized, baseline-corrected, smoothed, and aligned spectra of two milk tank samples: Valtierra (VTR) tank sample above and Ilarregi (ILR) tank sample below. These spectra have been obtained in a range of 600–2000 Da mass range in positive ionization mode (MS + ) using a Bruker Autoflex mass spectrometer fitted with a MALDI source.

The generated spectrums were normalized, smoothed, and aligned with each other, generating spectrums such as those in Figure 3.

After processing the data, 248 peaks above the background noise and different from the matrix interferences were identified. The signal/noise ratio in all of the peaks that were screened was always greater than three. The peaks were tentatively assigned to the lipid species that could present an ionization with the mass/charge ratio detected, identifying species of phosphatidylcholines, phosphatidylserines, sphingomyelins, and glycerides.

### 2.4. Classification of the Milk Samples Based on Their Haptoglobin Concentration and Lipid Biomarkers

**Figure 4:**
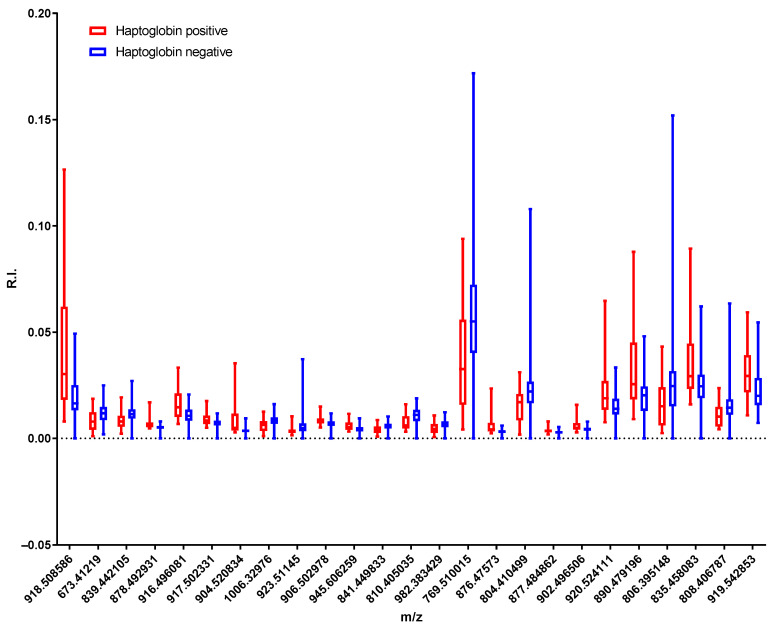
Box plot of the 25 most significant peaks of all of the samples found in the *t*-test. The box extends from the 25th to 75th percentiles and the whiskers go down to the smallest value and up to the largest.

Despite the large deviations between the values of some of the variables, the lipidomic analysis performed led to the identification of a set of mass channels that displayed significant differences between haptoglobin-positive and -negative milk. Figure 4 shows a box plot with the differences between the 25 most significant peaks.

**Figure 5:**
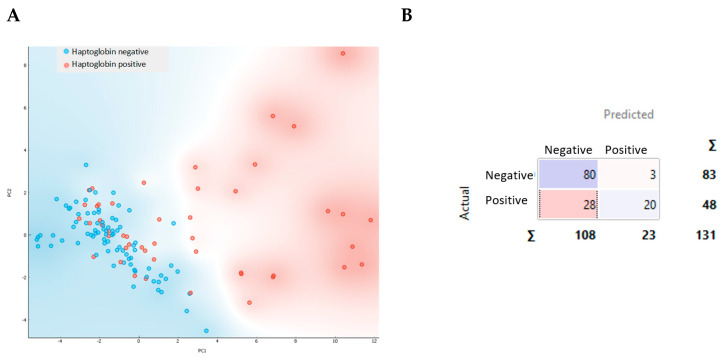
Principal component analysis with the projection of components one and two for the samples according to haptoglobin results, in red for positive and in blue for negative (**A**), and the confusion matrix obtained (**B**).

Principal component analysis (PCA) was performed using the 25 most significant peaks as variables in order to discriminate between the positive samples in the haptoglobin test and the negative ones. These peaks were selected based on the level of significance as a result of a Student’s *t*-test for this sample set (Table A4). The distribution generated by the PCA is shown in Figure 5.

**Figure A1:**
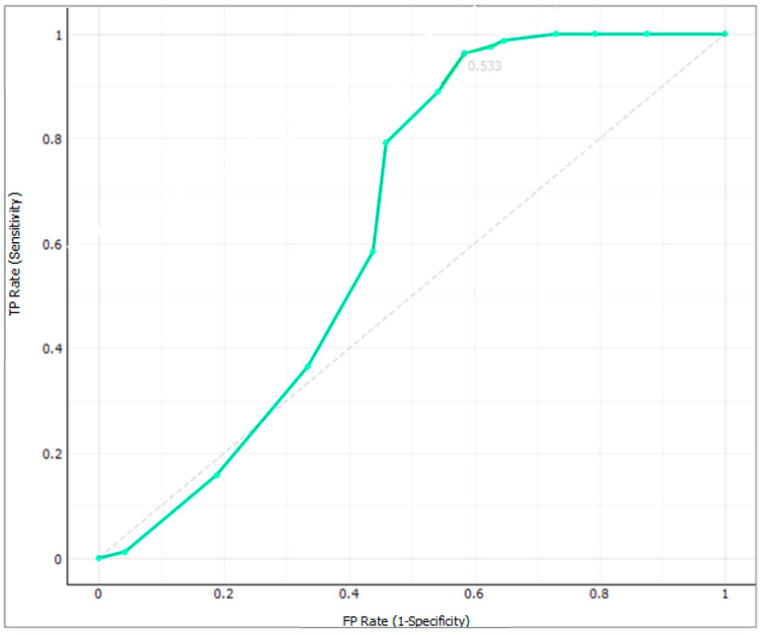
ROC curve of kNN for the haptoglobin class (-).

The negative resultant samples for the haptoglobin test were clustered in the region between the coordinates (-2,-2) and (4,4). Samples with less than 2 µg/mL haptoglobin in milk were in this range, while most of the positive samples were found at higher values. PCA revealed that this group of positive samples are clearly different from the negative ones (Figure 5A). This finding was also observed when using a k-nearest neighbors (kNN) classifier, which achieved an accuracy of 78.8% and a Recall of 76.3% (Figure 5B and Figure A1).

## 3. Discussion

The presence of the acute phase protein haptoglobin in blood and milk has been frequently associated with different pathologies in cows, such as mastitis, with the haptoglobin considered as a non-specific marker of stress, inflammation, or disease [18,20]. Subclinical cases of mastitis are responsible for the most of the economic losses associated with this pathology [36,37,38,39]. In this regard, a positive result in the Haptokit test was obtained for two of the nine farms under study. In the case of Fitero farm, this could be due to the serious mastitis problems it was suffering with several sick cows (whose milk was not collected in the tank) and numerous animals that had recently overcome infections at the time of sampling. Therefore, it is possible that among the herd there would have been animals in an initial phase of the development of mastitis as a result of transmissibility between animals that were sharing facilities and generating a concentration of haptoglobin in the tank. Thus, early identification of pathologies in asymptomatic stages can significantly contribute to reducing these burdens by improving the economic situation of the farmers and the welfare of the animals. The decision-making that farmers have to make daily in the management of their animals is a highly relevant factor in the economic impact of pathological processes [40]. Therefore, the possibility of having information that backs up and supports decision-making is of great value, since it could be applied to reduce the period in which the animal is not productive and diminish the number of disease courses suffered by each animal and the treatment time in each of them. All of this incurs a positive effect for the welfare of the animals. 

For this reason, the availability of biomarkers and assays to identify these situations or animal states has become one of the hot lines in the field. In this sense, the analysis of lipid biomarkers performed in this study clearly identified a cluster of haptoglobin-positive samples demonstrating the power of this technique to detect animals under acute stress. This methodology can be combined with microarrays to determine lipid fingerprints in a standardized and fast way providing a robust analysis that can be used to identify lipid-to-lipid or even protein-to-lipid relationships [41]. Lipidomic analyses has been widely used to study and also to identify lipidic biomarkers in several cancers such as melanoma or diseases and psychopathologies such as motivational deficits [42,43]. Moreover, dietary supplementation with certain lipids at the right time may prevent some of the symptoms occurring. For example, n-3 PUFA supplementation starting at birth, but not at weaning, seems to counteract the motivational deficits observed in mice with developmental deficiency in n-3 PUFA lipid species [42].

In the present study, the lipidomic analysis of cow’s milk samples performed by MALDI mass spectrometry revealed a set of biomarkers that strongly correlate with the presence of the acute phase protein haptoglobin. Phosphatidylcholines [PC_34:2], [PC_36:2] and [PC_36:3] were positively correlated with haptoglobin concentration, while [PC_28:0] and [PC_30:0] were negatively correlated. According to the bibliography on cow’s milk, the PC species [PC_28:0], [PC_30:0], [PC_34:2], [PC_36:2], and [PC_36:3] that correlate with haptoglobin concentration could correspond to PC (14:0/14:0), PC (16:0/14:0), PC (C16:0/C18:2), PC (C18:1/C18:1), and PC (C18:1/C18:2), respectively [44]. These data suggest that the presence of acute stress evoked a substitution of short-chain saturated phosphatidylcholines by longer-chain unsaturated ones in the cow’s milk that could anticipate prepathological stages. In this regard, Liu and coworkers describe in a lipidomic study performed on heat-stressed cow’s milk that TAG groups containing predominantly short- and medium-chain fatty acids were substituted by those containing mainly long-chain fatty acids, while the level of some polar lipid classes, especially LPC, were significantly reduced [16]. Heat stress can also be involved in our results as all the extractions of the samples were taken during the summer season, with maximum temperatures above 40 °C in the surroundings of the Ebro Valley. In fact, the concentrations of PCs that could be affected by heat stress in our results could correspond to PC (14:0/14:0), PC (16:0/14:0), PC (16:0/16:1), PC (16:0/18:2), PC (18:1/18:2), and PC (18:1/18:1) [16]. Different authors have indicated along the years that cattle are very sensitive to weather conditions, especially temperature and humidity [45,46,47,48]. High temperatures cause thermal stress in animals, which, when prolonged over time, has physiological effects and even clinical symptoms that can be seen with the naked eye [46]. Therefore, it is expected that one of the reasons why high haptoglobin levels were found in a significant percentage of healthy animals is the thermal stress to which animals were subjected during the dates on which the samples were taken [49]. Some previous studies have also proposed that exposure to heat stress can affect the milk fatty acid (FA) profile. In that sense, the abovementioned authors, Liu and coworkers, supplemented their lipidomic study with a global FA profile in a reduced cow’s cohort [16]. They described an increase in the content of long chain fatty acids (LCFA) accompanied by a reduction in short- and medium- chain fatty acids, similarly to other studies which also strictly controlled diet composition and feed [50] while others noted changes in unsaturated FA [51]. A limitation of the present research might be not including the milk FA profile, although some information can be obtained from a recent study [13]. These authors conducted a comprehensive analysis of the milk FA profile performed on a large number of cows (*n* = 297) and found no significant association between milk fatty acid (FA) and measured plasma haptoglobin levels [13]. In view of controversial outcomes, further experiments could be proposed in order to clarify these variations and other factors involved.

Another hypothesis that justifies the results obtained is that the animals classified as healthy were found to be suffering from a subclinical pathology without apparent symptoms, either because it was in the first phases or because it was a subclinical course. The prevalence of subclinical mastitis around the world is around 42%. Regarding the continental analysis, a high prevalence of subclinical mastitis was indicated in North America (46%), in Africa (44%)m and in Asia (42%). However, the prevalence of this subclinical pathology is lower in Europe (37%), in Oceania (36%), and in Latin America (34%) [52]. Despite not having official records or studies in Spain on the prevalence of diseases or injuries in dairy cattle farms, studies carried out in other countries are indicative. For example, in Finland, although there has been a sustained decline in the prevalence of subclinical mastitis over the last few decades, it endures at 19% [53]. Therefore, it is likely that a certain percentage of animals with a positive result for haptoglobin in milk is in a subclinical process.

Thus, the combination of acute stress haptoglobin with the lipid species identified in this study could be of great interest as a pathology marker in dairy cattle, allowing diagnosis to be anticipated after the development of symptoms and allowing the evolution of a disease to be monitored through specific biomarkers. This action would reduce non-productive periods and minimize treatments with a subsequent reduction in the associated economic losses [30]. 

## 4. Materials and Methods

### 4.1. Samples

Cow’s fresh milk samples were taken from nine different farms in the north of Spain: five farms from Navarra and four from Aragón. In all of the farms from Navarra, milk samples were taken from five healthy or pathological cows that were in the herd and were part of the milking rotation. At the same time, in some of these Navarrese farms, samples also were taken from some of the animals separated from the herd, which were suffering from an inflammatory process. A total of 25 milk samples were directly taken from the udders of supposedly healthy cows and a total of 14 milk samples from those in pathological process, collecting in both cases a volume of 50 mL. In addition, at the nine farms, samples were also taken from milk storage tanks after milking of the cows. The milking of the cows was carried out between 24 and 48 h before sampling; for this reason they were considered representative samples of the farm. All of the samples were frozen and kept at −20 °C until processing.

During sampling, information on the farm and the animals was collected, such as the location and size (number of cattle) of the farm, the feed of the animals, the Welfare Quality^®^ score or rating from the last audit, the farm’s internal registration tag number, the age (years/months) of the animals, the number of lactations of the animals, and the type of pathology in the case of sick animals.

At the time of processing, samples were thawed at room temperature to prevent thermal damage to haptoglobin and coagulation of fat globules. 

### 4.2. Haptokit

Haptokit is a sandwich-type immunoassay developed by the company IMG Pharma Biotech S.L. (Derio, Spain) which aids in the detection of haptoglobin through its specific binding to two antibodies at two different epitopes of the protein, with the limit of detection being 3 µg/mL.

**Figure 6:**
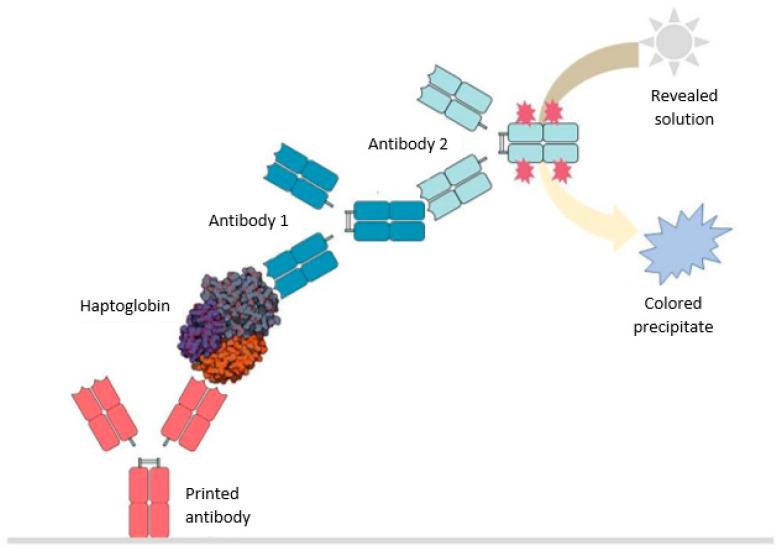
Scheme of haptoglobin detection through the immunoassay developed *in house* at IMG Pharma Biotech S.L. for its use in fresh milk.

The primary antibody is printed on a solid surface on which the incubation of the milk without previous treatment takes place (Figure 6). Subsequently, it is washed in a saline solution to remove the milk and it is incubated again with the antibody mixture. The second incubation solution is prepared prior, and in it, the union of the two antibodies takes place which allows for the generation of a colored precipitate observable with the naked eye: anti-haptoglobin antibody 1 and anti-antibody 1 antibody 2. Antibody 2 is the one with a molecule linked to the heavy chain that reacts with the development solution generating a blue precipitate proportional to the amount of protein that has been retained by the printed antibody.

After incubation of the antibody mixture, another wash is performed in a saline solution to completely remove the antibodies that have not been retained by binding to haptoglobin, and this may generate a nonspecific signal. Finally, a final incubation process is carried out with the development solution that will cause the generation of the blue precipitate which will be considered as a positive result.

**Figure 7:**
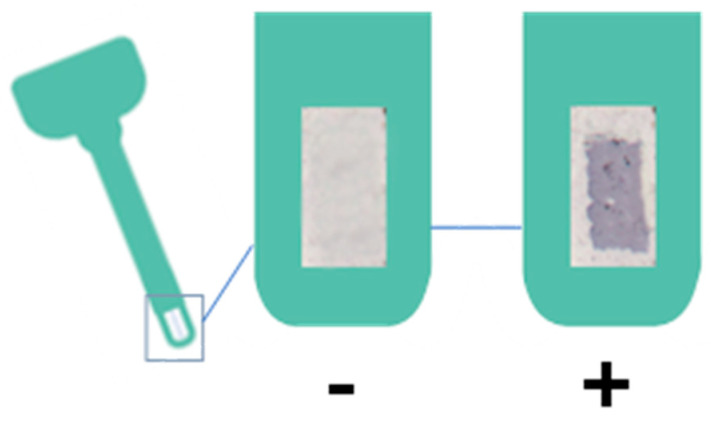
Negative and positive result of the haptoglobin test.

The test will be considered positive when the three points in which the blue-colored antibodies have been printed can be seen with the naked eye, with it normally being more comfortable to observe on a light-colored background (Figure 7). The results have been scanned in order to be able to compare the coloration under the same conditions, on the same background, and just after performing the test.

### 4.3. MALDI-TOF Mass Spectrometry

Sample analysis was performed using an Autoflex III model mass spectrometer (Bruker, Billerica, MA, USA). This equipment has a solid-state laser that emits ultraviolet light at 355 nm. An average of 3000 shots per spectrum were made, avoiding the deposits of caseins and proteins that prevented the formation of crystals. Spectrums were acquired in the *m/z* interval from 600 to 2000 Da, the area in which lipids are expected to be found. The equipment has a flight tube as a mass analyzer, together with a multichannel plate (MCP) as an ion detector.

In this study, the “shotgun” strategy was followed for the application of spectrometry in the field of lipidomics. In this approach, the mass spectrum forms a lipid fingerprint of the analyzed sample, in which individual molecular species of most major and many minor lipid classes can be directly quantified without the need of chromatographic purification. This strategy is fast and highly sensitive and can identify hundreds of lipids missed by other methods using smaller amounts of sample; it is very useful to first determine differences in the lipid composition of the samples before identifying the species responsible for these changes. The objective of applying this technique is to observe variations in the lipid profiles of the distinct samples analyzed and to assess the similarities by applying different criteria for their grouping.

2-mercaptobenzothiazole (MBT) was used as a matrix for ionization in positive mode, with this being an ideal matrix for the detection of lipids that favor their ionization and presenting high stability under high vacuum conditions, an essential requirement for mass spectrometry.

The matrix was prepared in saturation in a buffer (IMG Pharma Biotech S.L.’s own development) that allows both optimal co-crystallization and ionization. The sample was diluted in the matrix solution 1:5 *v/v* (sample/matrix) performing a gentle homogenization to be later deposited on the conductive plate for analysis in the spectrometer.

A volume of 2 µL of the sample/matrix solution was deposited, allowing for crystallization by evaporation at room temperature. Each sample was analyzed in duplicate, including a matrix solution as a blank in order to remove interferences due to its ionization.

A Thermo LTQ Orbitrap XL mass spectrometer was used for fragmentation. An extract was prepared by mixing the three milk samples and the IPA protocol described by Iriondo et al. in 2019 [54] was used for lipid extraction. Once the lipid extraction was prepared, the MBT matrix was used as it was used when analyzing the samples in the Autoflex III model mass spectrometer (Bruker, Billerica, MA, USA). Firstly, a tentative identification of all the lipids detected in the full scan MS- was made and the peak corresponding to [PC 36:3–H]- was fragmented to confirm that the species were present in the milk and that the chains that compose it coincide with those of the article written by Liu et al. [34]. For this, a CID (collision-induced dissociation) with a collision energy of 90 was used.

### 4.4. Spectrum Processing

All the data generated in the mass spectrometer were processed through different computer applications developed by IMG Pharma Biotech S.L. using Matlab (MathWorks, Natick, MA, USA) as the main programming language. Before processing the data, all of the generated spectrums were exported from the files generated by the spectrometer and transformed into a format (.txt) in order to be able to work with the data in the previously mentioned applications.

Once the data were exported to .txt format, baseline correction was performed in order to adequately determine the intensity of the peaks. Alignment of all of the spectra was also performed by the method of correlation to the average spectrum. Subsequently, the data were smoothed using the Savitzky Golay algorithm for noise removal. Finally, normalization to total ionic current was performed and peaks with an S/N (signal/noise) ratio greater than three were selected.

The selected peaks were assigned by a database search using an algorithm owned by IMG Pharma Biotech S.L. in order to determine the lipid species corresponding to each *m/z*, taking into account the different ionizations they undergo.

### 4.5. Statistical Analyses

The homogeneity of the variances of the animals classified as healthy and pathological, and those that were positive in the haptoglobin detection test in milk versus the negative ones, was evaluated through the Fisher’s F test. On the other hand, to identify the peaks that present significant differences between the means of the healthy and pathological cows and, on the other hand, the positive and negative cows to the haptoglobin detection test in milk, *t*-test statistical analysis was used. Levene’s test was used to evaluate the hypothesis that the variances in the independent samples are similar, and its result allowed for validation of the possibility that they could be compared and considered as significant differences using the one-factor ANOVA test. One-factor analysis of variance was performed in cases where more than two groups were available (identification of the most relevant peaks to discern between the farms of origin of the samples). Pearson’s correlation coefficient was used to assess the correlation between the milk tanks of the farms and the respective samples of individual animals classified based on their status as pathological animals or healthy animals. In all of the statistical analyses, the confidence level used was 95%. All of the statistical analyses were performed using SPSS 26.0 (IBM, Endicott, NY, USA), Orange Biolab (University of Ljubljana), and Matlab.

### 4.6. Lipid Assignment

**Figure A2:**
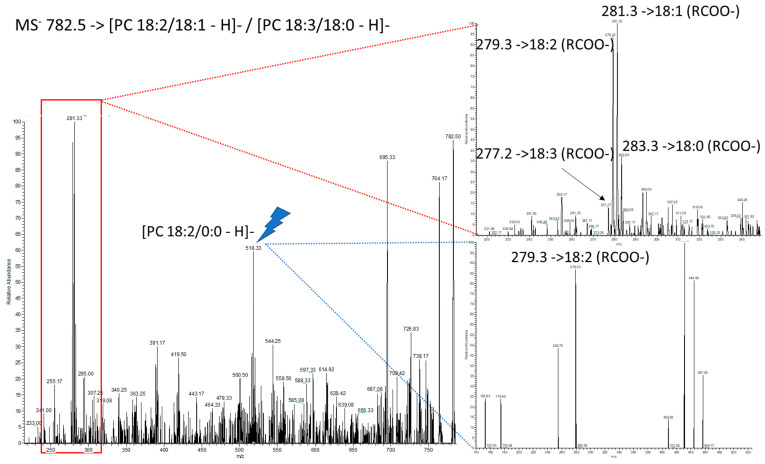
MS/MS and MS^3^ analysis of the extract of the mixture of the three different types of milk with MALDI on the *m/z* corresponding to the adduct [PC 36:3 -H]- (782.5).

A tentative data assignment was made using a synthetic database and the Lipid Maps database. Some of the species found were confirmed using the database from the article written by Liu et al. on bovine milk [34]. To confirm that the lipid species identified in the article written by Liu et al. corresponded to those of the analyzed milk samples, MS/MS and MS^3^ analysis with MALDI was performed on the *m/z* corresponding to the adduct [PC 36:3-H]-(*m/z* 782.5), thus verifying its presence in the sample and that the majority of the isomers coincide with those indicated by Liu et al. in 2020 [34] (Figure A2).

## Figures and Tables

**Table 1:** List of the seven peaks that present significant correlations with haptoglobin, its lipid assignment, and their isomers. The species found in this table are ionized with Cs.

*m*/*z*	Lipid	Isomer I *	Isomer II *	Isomer II *	Correlation
902.54	HexCer d39:1	d16:1/23:0	d17:1/22:0		0.84
903.52	SM d39:2	d16:1/23:1			0.57
916.48	PC 36:3	18:1/18:2	18:0/18:3	16:0/20:3	0.51
973.61	SM d44:2	d18:1/26:1			−0.46
706.33	HexCer d25:1	NM			−0.48
825.44	SM t32:0	NM			−0.48
864.44	PC 32:1	18:1/14:0	16:0/16:1	17:1/15:0	−0.55

* Confirmed lipid species by UHPLC-HESI MS^2^. Adapted with permission from Ref. [34]. Copyright 2020, American Chemical Society. NM: no match.

**Table 2:** Haptoglobin detection results in the milk tank samples.

Location of Farms	Farm Name	Reference Name of the Farm	Results in the Haptokit Test
Aragón	Alfajarín	ALF	+
	Montañana	MNT	−
	Miralbueno	MRB	−
	Movera	MVR	−
Navarra	Marcilla	MAR	−
	Valtierra	VTR	−
	Murchante	MCH	−
	Fitero	FTR	+
	Ilarregi	ILR	−

## Data Availability

The data presented in this study are available on request from the corresponding author. The data are not publicly available due to IPR.

## Appendix A

**Table A1:** Milk control samples owned by IMG Pharma whose haptoglobin concentration was determined by ELISA.

Milk Sample	Haptoglobin Concentration (µg/mL)
1 green	14.71
2 green	2.19
3 green	89.2
1 blue	1.81
2 blue	0.37
3 blue	56.4
1 red	252.0
2 red	225.6
3 red	48.2
Control	2.86

**Table A2:** Individual sample results for haptoglobin detection (>2 μg/mL) with the Haptokit test.

Animal Type	Farm’s Name	Samples	Results of the Haptokit Test	Prevalence of Positive Results in Each Animal Group
Healthy cows	Marcilla farm	1	−	
		2	+	
		3	−	
		4	+	
		5	−	
	Valtierra farm	6	−	
		7	−	
		8	−	
		9	−	
		10	−	
	Murchante farm	11	−	24%
		12	−	
		13	−	
		14	−	
		15	+	
	Fitero farm	16	-	
		17	−	
		18	−	
		19	+	
		20	+	
	Ilarregi farm	21	-	
		22	+	
		23	−	
		24	−	
		25	−	
Pathological cows	Marcilla farm	1	+	50%
		2	−	
		3	−	
	Valtierra farm	4	+	
	Murchante farm	5	+	
		6	−	
	Fitero farm	7	+	
		8	−	
		9	+	
		10	+	
	Ilarregi farm	11	−	
		12	+	
		13	−	
		14	−	

**Table A3:** Summary of the data obtained from the farms on which the samples were taken.

Farm’s Name	Reference	Healthy Animal Samples	Pathological Animal Samples	Tank’s Samples	Nº of Cattle	More Recent Welfare Quality^®^ Qualification	Feed	Observations
Alfajarín	ALF	-	-	1	70	GOOD	-	Full tank of two milking batches.
Montañana	MNT	-	-	1	50	ENOUGH	-	Full tank of two milking batches.
Miralbueno	MRB	-	-	1	60	GOOD	-	Full tank of two milking batches.
Movera	MVR	-	-	1	120	GOOD	-	Full tank of two milking batches.
Marcilla	MAR	5	3	1	90	ENOUGH	12 kg feed + 10 kg alfalfa silo + 12 kg ray grass silo + 4 kg fescue silo + 4 kg pea silo + 0.6 kg straw	Samples collected during afternoon milking. Temperatures above 40 °C.
Valtierra	VTR	5	1	1	105	GOOD	11 kg feed + 10 kg silo ray grass + 13 kg silo corn + 10 kg pulp beetroot + 1 kg straw + 4 kg alfalfa silo	Samples collected during afternoon milking from a single batch. Heat and storm during the visit.
Murchante	MCH	5	2	1	260	ENOUGH	-	Samples taken during morning milking with the tank full from two milking runs. Sick animals have their own stables.
Fitero	FTR	5	4	1	90	GOOD	12 kg feed + 10 kg ray grass silo + 26 kg corn silo	Tank sample from two milking batches, collected on an afternoon with temperatures above 40 °C. Farm with mastitis problems, active epidemic, and with numerous affected animals throughout the summer.
Ilarregi	ILR	5	4	1	30	GOOD	10 kg feed + 15 kg silo local grass + sheepherding at discretion	Animals with freedom and sheepherding on hills, which has caused them to be subject to different accidents. Above all, limps and back injuries due to the interaction between them. Sampling during the morning and with the tank full from milking.

**Table A4:** Mass channels showing significant differences between negative and positive values for haptoglobin. The relative magnification is (intensity (+)−intensity (-))/intensity (-). The species found in this table are ionized with Cs.

*m*/*z*	Lipid #	Issomer I #	Issomer II #	Issomer III #	Issomer IIII #	Bartlett *p* Value	Mean Comparison Method	*p* Value	Sig	Relative Increase
673.4122	NM					0.9043	T-test (Parametric)	0.0000	****	−0.340
677.3009	NM					0.5619	*t*-test (Parametric)	0.0006	***	−0.230
722.3078	NM					0.0382	Wilcoxon (Non-parametric)	0.0056	**	−0.239
728.5111	NM					0.0016	Wilcoxon (Non-parametric)	0.0175	*	−0.188
764.5491	NM					0.0607	*t*-test (Parametric)	0.0095	**	−0.176
769.51	NM					0.0359	Wilcoxon (Non-parametric)	0.0003	***	−0.361
781.4299	NM					0.0000	Wilcoxon (Non-parametric)	0.0234	*	−0.193
792.5595	NM					0.2846	*t*-test (Parametric)	0.0214	*	−0.145
804.4105	PE-P 32:2	14:0p/18:2	16:1p/16:1			0.0000	Wilcoxon (Non-parametric)	0.0002	***	−0.350
806.3951	PE-P32:1	14:0p/18:1	16:0p/16:1			0.0000	Wilcoxon (Non-parametric)	0.0002	***	−0.426
807.422	NM					0.3173	*t*-test (Parametric)	0.0053	**	−0.192
808.4068	PC 28:1	14:0/14:1	10:0/18:1	10:0/18:1	16:1/12:0	0.0000	Wilcoxon (Non-parametric)	0.0002	***	−0.328
809.46	SM 32:0	d16:0/16:0	d18:0/14:0			0.2844	*t*-test (Parametric)	0.0016	**	−0.177
810.405	PC 28:0	16:0/12:0	14:0/14:0			0.2296	*t*-test (Parametric)	0.0001	****	−0.275
821.4216	SM 33:1	d17:1/16:0	d18:1/15:0			0.7195	*t*-test (Parametric)	0.0360	*	−0.098
835.4581	SM 34:1	d16:1/18:0	d18:1/16:0			0.0001	Wilcoxon (Non-parametric)	0.0002	***	0.423
836.469	NM					0.0000	Wilcoxon (Non-parametric)	0.0008	***	0.286
837.4799	SM 34:0	d18:0/16:0	d16:0/18:0			0.0001	Wilcoxon (Non-parametric)	0.0146	*	0.170
838.43	PC 30:0	16:0/14:0				0.9789	*t*-test (Parametric)	0.0010	**	−0.250
839.4421	NM					0.9406	*t*-test (Parametric)	0.0000	****	−0.293
841.4498	NM					0.1850	*t*-test (Parametric)	0.0000	****	−0.253
843.4841	NM					0.4795	*t*-test (Parametric)	0.0008	***	−0.218
845.4523	NM					0.8644	*t*-test (Parametric)	0.0316	*	−0.118
851.4923	NM					0.0000	Wilcoxon (Non-parametric)	0.0482	*	−0.169
865.4501	NM					0.7561	*t*-test (Parametric)	0.0103	*	−0.098
876.4757	PC-P 34:1	16:0p/18:1				0.0000	Wilcoxon (Non-parametric)	0.0000	****	0.803
877.4849	NM					0.3720	*t*-test (Parametric)	0.0001	****	0.280
878.4929	NM					0.0000	Wilcoxon (Non-parametric)	0.0000	****	0.430
879.478	NM					0.4485	*t*-test (Parametric)	0.0253	*	0.121
890.4792	PE 37:2	19:1/18:1				0.0000	Wilcoxon (Non-parametric)	0.0004	***	0.729
891.4703	NM					0.0008	Wilcoxon (Non-parametric)	0.0033	**	0.328
892.4964	NM					0.7175	*t*-test (Parametric)	0.0023	**	0.263
893.4884	NM					0.6588	*t*-test (Parametric)	0.0178	*	0.188
902.4965	PC-P 36:2	18:0p/18:2				0.0000	Wilcoxon (Non-parametric)	0.0001	****	0.433
904.5208	PC-P 36:1	18:0p/18:1				0.0000	Wilcoxon (Non-parametric)	0.0000	****	1.357
906.503	PC 35:1	17:0/18:1	17:1/18:0	19:1/16:0		0.2284	*t*-test (Parametric)	0.0000	****	0.213
908.5696	NM					0.6146	*t*-test (Parametric)	0.0415	*	−0.099
911.596	NM					0.0654	*t*-test (Parametric)	0.0337	*	−0.179
916.4961	PC 36:3	18:1/18:2	18:0/18:3	16:0/20:3		0.0000	Wilcoxon (Non-parametric)	0.0001	****	0.530
917.5023	NM					0.0007	Wilcoxon (Non-parametric)	0.0002	***	0.344
918.5086	PC 36:2	18:1/18:1	18:0/18:2			0.0000	Wilcoxon (Non-parametric)	0.0000	****	1.284
919.5429	SM 40:1	d16:1/24:0	d17:1/23:0	d18:1/22:0		0.0009	Wilcoxon (Non-parametric)	0.0003	***	0.396
920.5241	PC 36:1	18:0/18:1				0.0000	Wilcoxon (Non-parametric)	0.0001	***	0.599
921.5324	NM					0.0184	Wilcoxon (Non-parametric)	0.0009	***	0.299
922.5318	NM					0.4771	*t*-test (Parametric)	0.0058	**	0.142
923.5115	NM					0.0000	Wilcoxon (Non-parametric)	0.0000	****	−0.412
925.504	NM					0.2278	*t*-test (Parametric)	0.0082	**	−0.153
945.6063	SM 42:2	d18:1/24:1				0.0125	Wilcoxon (Non-parametric)	0.0000	****	0.380
946.5717	NM					0.0000	Wilcoxon (Non-parametric)	0.0002	***	0.585
957.558	NM					0.0010	Wilcoxon (Non-parametric)	0.0199	*	−0.197
961.587	NM					0.2321	*t*-test (Parametric)	0.0140	*	−0.171
962.5731	NM					0.0021	Wilcoxon (Non-parametric)	0.0307	*	−0.170
979.516	NM					0.2131	*t*-test (Parametric)	0.0287	*	−0.167
980.3565	NM					0.8849	*t*-test (Parametric)	0.0005	***	−0.239
982.3834	NM					0.4531	*t*-test (Parametric)	0.0001	****	−0.277
1006.3298	NM					0.4139	*t*-test (Parametric)	0.0000	****	−0.265
1009.4115	NM					0.9389	*t*-test (Parametric)	0.0017	**	−0.189
1010.3477	NM					0.7787	*t*-test (Parametric)	0.0066	**	−0.165
1070.4714	NM					0.0026	Wilcoxon (Non-parametric)	0.0278	*	−0.196
1093.3747	NM					0.2474	*t*-test (Parametric)	0.0072	**	−0.225
1119.465	NM					0.3233	*t*-test (Parametric)	0.0350	*	−0.272
1128.4544	NM					0.0882	*t*-test (Parametric)	0.0488	*	−0.177
1130.4393	NM					0.3415	*t*-test (Parametric)	0.0058	**	−0.273
1138.4385	NM					0.0190	Wilcoxon (Non-parametric)	0.0063	**	−0.249
1147.4911	NM					0.0128	Wilcoxon (Non-parametric)	0.0116	*	−0.235
1151.5643	NM					0.0000	Wilcoxon (Non-parametric)	0.0052	**	−0.466
1175.9367	NM					0.0342	Wilcoxon (Non-parametric)	0.0296	*	−0.202
1178.7439	NM					0.0071	Wilcoxon (Non-parametric)	0.0151	*	−0.270
1189.9013	NM					0.0055	Wilcoxon (Non-parametric)	0.0370	*	−0.296
1206.6016	NM					0.3981	*t*-test (Parametric)	0.0360	*	−0.259
1261.6735	NM					0.0000	Wilcoxon (Non-parametric)	0.0060	**	−0.377
1384.1603	NM					0.0000	Wilcoxon (Non-parametric)	0.0002	***	−0.622

(*) *p*-value <0.05; (**) *p*-value <0.01; (***) *p*-value <0.001; (****) *p*-value <0.0001. # Confirmed lipid species by UHPLC-HESI MS^2^. Adapted with permission from Ref. [34]. Copyright 2020, American Chemical Society. NM: no match.
